# A best-practice position statement on pregnancy after kidney transplantation: focusing on the unsolved questions. The Kidney and Pregnancy Study Group of the Italian Society of Nephrology

**DOI:** 10.1007/s40620-018-0499-x

**Published:** 2018-06-14

**Authors:** Gianfranca Cabiddu, Donatella Spotti, Giuseppe Gernone, Domenico Santoro, Gabriella Moroni, Gina Gregorini, Franca Giacchino, Rossella Attini, Monica Limardo, Linda Gammaro, Tullia Todros, Giorgina Barbara Piccoli, The Kidney and Pregnancy Study Group of the Italian Society of Nephrology

**Affiliations:** 1 Azienda Ospedaliera Brotzu, Cagliari, Italy; 2 IRCCS San Raffaele Hospital, Milan, Italy; 3 Santa Maria Degli Angeli Hospital, Putignano, Italy; 4 Azienda Ospedaliera Universitaria G. Martino, Messina, Italy; 5 Fondazione Ca’ Granda Ospedale Maggiore, Milan, Italy; 6 Spedali Civili di Brescia, Brescia, Italy; 7 Ospedale d’Ivrea, Ivrea, Italy; 8 Department of Surgery, Università di Torino, Turin, Italy; 9 Azienda Ospedaliera della Provincia di Lecco, Lecco, Italy; 10 Ospedale Fracastoro, San Bonifacio, Italy; 11 Department of Clinical and Biological Sciences, Università di Torino, Turin, Italy; 12 Centre Hospitalier Le Mans, Le Mans, France

**Keywords:** Chronic kidney disease, Evidence-based medicine, Pregnancy, Hypertension, Proteinuria, Preeclampsia, Pre-term delivery

## Abstract

Kidney transplantation (KT) is often considered to be the method best able to restore fertility in a woman with chronic kidney disease (CKD). However, pregnancies in KT are not devoid of risks (in particular prematurity, small for gestational age babies, and the hypertensive disorders of pregnancy). An ideal profile of the potential KT mother includes “normal” or “good” kidney function (usually defined as glomerular filtration rate, GFR ≥ 60 ml/min), scant or no proteinuria (usually defined as below 500 mg/dl), normal or well controlled blood pressure (one drug only and no sign of end-organ damage), no recent acute rejection, good compliance and low-dose immunosuppression, without the use of potentially teratogen drugs (mycophenolic acid and m-Tor inhibitors) and an interval of at least 1–2 years after transplantation. In this setting, there is little if any risk of worsening of the kidney function. Less is known about how to manage “non-ideal” situations, such as a pregnancy a short time after KT, or one in the context of hypertension or a failing kidney. The aim of this position statement by the Kidney and Pregnancy Group of the Italian Society of Nephrology is to review the literature and discuss what is known about the clinical management of CKD after KT, with particular attention to women who start a pregnancy in non-ideal conditions. While the experience in such cases is limited, the risks of worsening the renal function are probably higher in cases with markedly reduced kidney function, and in the presence of proteinuria. Well-controlled hypertension alone seems less relevant for outcomes, even if its effect is probably multiplicative if combined with low GFR and proteinuria. As in other settings of kidney disease, superimposed preeclampsia (PE) is differently defined and this impairs calculating its real incidence. No specific difference between non-teratogen immunosuppressive drugs has been shown, but calcineurin inhibitors have been associated with foetal growth restriction and low birth weight. The clinical choices in cases at high risk for malformations or kidney function impairment (pregnancies under mycophenolic acid or with severe kidney-function impairment) require merging clinical and ethical approaches in which, beside the mother and child dyad, the grafted kidney is a crucial “third element”.

## Introduction: an historical note

The history of pregnancy after kidney transplantation starts with young twin sisters: “In May, 1956, one of a pair of 21-year-old identical twin females from Oklahoma was being studied as a potential recipient for a kidney transplant from her twin sister”. It is interesting to note that in this paper the family status was reported in the opening sentences: “Both were childless, having been married for less than a year.” The state of the recipient was defined as “dire” “with hypertension (blood pressure of 190 systolic, 120 diastolic), congestive heart failure (…)” [1].

The paper reports that this woman regained her health, recovered her menstrual cycle and soon became pregnant, giving birth, by caesarean section, chosen for fear of renal compression, to a healthy baby. The donor and recipient were enthusiastic enough to give birth, as the paper reports, to a total of five babies, all of them in good health, at the time The New England Journal of Medicine paper was published [1].

In 1966, Murray et al. wrote in JAMA: “Medical advances force new judgments and evaluations. The ethics, legality, and morality of organ transplantation, a recent and still unpredictable therapeutic procedure, has already elicited editorials, special articles, and conferences (…)”, underlining the importance of maintaining what we now call quality of life, which was implicit in the job of a good physician, before measurement was attempted.

In this context it is worth quoting the paper entitled “Transplantation and haemodialysis. The recipient’s response to renal transplantation”, in which we read: “I guess, like other people, I never fully appreciated the simple things in life until I lost them. Just to be able to get up in the morning and go out and do a day’s work is a wonderful feeling (…)” [2].

When Edith Helm died, 55 years after having transplantation, leaving a son, a daughter, four grandchildren and four great-grandchildren, Professor Murray, who considered the sisters and their families part of “the extended Murray family”, thanked her for her contribution to demonstrating that having a child after transplantation was possible. Besides eliciting admiration for the Nobel Prize winner, this story should also teach us the importance of curiosity and humility, and of personalised solutions in the management of pregnancy after kidney transplantation [3]. Not many years would pass before there were reports of successful pregnancies after transplantation from non-identical twins and of vaginal deliveries [4, 5]. The era of pregnancy after kidney transplantation had begun [6–8].

## Evidence-based medicine and pregnancy after KT: methodological insights

Evaluating the evidence on pregnancy in kidney transplantation (KT) is dogged by many of the same methodological problems as that on pregnancy in chronic kidney disease (CKD) or in patients on dialysis [9–16]. A common language is lacking, in particular for the definition of preeclampsia (PE), and for preterm delivery. The population is highly heterogeneous, and relatively few large studies are available; the periods of study are different and stratification is seldom attempted. Information on miscarriages is scant, and is usually limited to small single-centre series, due to the difficulties inherent in tracking the data on larger series or registries [17–26]. Hence, most systematic reviews and large series conclude by stating that more evidence is needed, and while we have data on “ideal patients”, less is known about patients with reduced kidney function, disease recurrence or proteinuria.

After kidney transplantation, as in all the CKD stages, the degree of kidney impairment, hypertension and proteinuria are acknowledged factors in the pathogenesis of adverse pregnancy-related outcomes, even though their pathogenesis is incompletely understood [27–32].

Kidney function assessment in pregnancy is a challenge both after KT and in CKD: no validated formula exists and stratifications based upon serum creatinine, or serum cystatin-C are not commonly employed [33–43]. Because there are so few randomized controlled trials (RCTs) and large observational studies, we will deal mainly with low levels of recommendation and not graded suggestions, so that in this paper we present what constitutes “best practice”, underlining the key role experience plays in supporting clinical choices [9, 10].

## Evidence-based medicine and pregnancy after KT: bioethical aspects

(i) Pregnancy is not a “zero-risk” choice. Grading the risks of pregnancy after kidney transplantation is difficult, in particular in cases that differ from the “ideal” candidate (strong suggestion, evidence from different sources, mainly epidemiological studies).
(ii) Pregnancy after KT poses important ethical problems (strong suggestion, beyond evidence).
(iii) Pregnancy in “non-ideal” candidates after KT is an example of bioethical conflict, in which the principles of ethical assessment may give rise to contrasting evaluations (strong suggestion, beyond evidence).

Pregnancy is not a “zero-risk” situation: in the general population, more than 10% of pregnancies are complicated by hypertensive disorders (including 3–5% of PE) or by gestational diabetes [44–47]. All these events are more common in CKD, starting in its early stages, and this also applies to KT patients with normal kidney function [32, 48–51].

In the absence of a precise quantification of the risks across the scale of kidney function and proteinuria, it is difficult to define what an “acceptable risk” is for a pregnancy after KT [52–54]. In fact, with the excellent results of the first pregnancies after KT pregnancy became an option that gave rise to great expectations—the conviction that restoring fertility after a successful KT would be possible in a not too distant future [1, 2, 4–6, 55]. As will be discussed below, the ideal candidate for pregnancy after KT is a woman with normal kidney function, no proteinuria, normotension, no recent acute rejection, good compliance and low dose immunosuppression, who has not taken potentially teratogen drugs [mycophenolic acid and mammalian target of rapamycin (m-Tor) inhibitors] in the last months, and after at least 1–2 years after transplantation.

One of the main issues in this context is the definition of “normal” or “good” kidney function: the classic CKD classification is not routinely used after kidney transplantation, and studies use different cut-points as for definition of normal or good kidney function [17–26].

There is no doubt that a glomerular filtration rate (GFR) above 90 ml/min defines a normal renal function; “good” renal function is usually defined as a GFR above 60 ml/min, even if, at least according to the Italian experience, there may be significant differences between patients with GFR ≥ 90 ml/min and those with 60–90 ml/min of GFR [56].

Likewise, the definition of the cut-points for proteinuria and hypertension are non-univocal; while there is no doubt that, as well as in the overall population, blood pressure level below 130/80 is optimal and proteinuria in pregnancy is considered as normal when below 300 mg/day, there is a grey zone for blood pressure level below 140/90 and proteinuria below 300–500 mg/day [17–26, 56].

The Study Group therefore proposes an individualised evaluation in all cases with GFR (at best assessed by creatinine clearance) below 90 ml/min, blood pressure above 130/80 mmHg and proteinuria above 300 mg/day.

The literature reports few cases of pregnancy in the context of malfunctioning KT, suggesting that counselling has systematically discouraged pregnancies in “less than ideal” conditions; therefore, risk assessment in a “failing” KT is presently difficult. Even if not quantified, these situations constitute a risk of developing severe hypertension, proteinuria and prematurity, and of accelerating kidney failure [6–8, 32]. Hence, in the presence of strong determination to have a baby, the clinical decision must merge with an ethical approach.

Pregnancy in the context of “less than ideal” KT is an example of conflict between four main principles of medical ethics (autonomy, non-maleficence, beneficence, and justice). The benefit for a KT woman of having a baby is clear, and conflicts with the potential harm to the kidney graft, to maternal health and to the baby, who risks being born preterm, with potential consequences for its long-term health. None of these risks is however clearly quantifiable. The concept of distributive justice, that considers KT as a resource in contrast with decisions that could accelerate its loss, while ensuring the patient’s autonomy means that the final choice cannot be taken by a third party [55–59].

The hierarchy of ethical principles differs from one country to another. In Mediterranean countries, where the patient-physician relationship historically followed a paternalist model, the principle of *primum non nocere* supported the negative medical attitude towards pregnancy in less-than-optimal conditions. Conversely, in English-speaking countries, where the patient’s self-determination is seen as paramount, autonomy comes first, and it is felt that it should be respected after ensuring that the patient has comprehended and accepted the risks her decision involves. The practice, observed in some transplant centres, of asking the patients to avoid pregnancy, as a way to optimize the social advantage of a successful transplantation, considers justice to be the leading principle [60–68].

There is no “right answer” in bioethics. However, the current ethical approaches, under the influence of Anglo-American bioethics, privilege autonomy, a concept that is increasingly integrated in juridical recommendations. The paradigm shift from paternalism to self-determination and from non-maleficence to autonomy will probably lead to a more permissive attitude towards pregnancy after KT in less than optimal conditions.

## What are the main adverse outcomes in pregnancy in KT patients?

(i) The risk of maternal death is low and difficult to quantify (strong suggestion; scattered evidence).
(ii) The risks of worsening kidney function are low in KT patients with normal kidney function, but have not been clearly quantified in other cases (moderate suggestion; scattered evidence).
(iii) The risks to the foetus are mainly related to prematurity (strong suggestion; evidence from large observational studies and registries).
(iv) Foetal malformations do not increase in KT patients, if teratogen drugs are avoided (strong suggestion, large body of evidence from different sources).
(v) There is no substantial difference in outcomes between patients treated with non-teratogen drugs; calcineurin inhibitors may be associated with risk of foetal growth restriction (strong suggestion; indirect evidence from different sources).
(vi) The risk of adverse maternal-foetal outcomes appears to be higher in multiple pregnancies (moderate suggestion, few available data).
(vii) The risk of adverse maternal-foetal outcomes appears to be higher in assisted-fertilization pregnancies (moderate suggestion, few available data).

Maternal death is rare enough to represent a non-quantifiable risk during or immediately after a KT pregnancy. The long-term risks are difficult to quantify and should probably be discussed in relation to pregnancy when counselling dialysis patients [16, 69–73].

The risk of kidney function impairment is low in KT patients who start pregnancy with normal kidney function. The definition of normal kidney function is however not univocal, and is often based on creatinine levels and not on creatinine clearance or GFR [38, 39, 43].

As will be discussed below, the risks are higher in patients with reduced kidney function; this should be obvious considering the progressive impairment of the results seen in non-KT patients with CKD [13, 28, 30, 74–76].

New onset of proteinuria or increase of pre-existing proteinuria seems to be common; once more, the lack of standard criteria and the low use of 24-h proteinuria after KT make it difficult to assess the evidence [77].

Prematurity (defined as birth before 37 completed gestational weeks), early preterm delivery (before 34 completed gestational weeks) and extremely preterm delivery (before 28 gestational weeks) increase in KT pregnancies and the increase is greater in KT patients with impaired kidney function. No graduation of the effect across stages is yet available.

Among the many risks linked to prematurity that should be addressed in counselling (perinatal death, retinopathy, and neurological problems) the possibility that premature babies are more likely to develop CKD and hypertension or cardiovascular diseases in adulthood should be discussed [78–94].

There is no evidence that children from KT mothers have a higher rate of malformations than the general population, except for those linked to genetic diseases, such as congenital abnormalities of the kidneys and of the urinary tract (CAKUT) or autosomal dominant polycystic kidney disease (ADPKD) [17–21, 23, 56, 95–100].

Data on CKD and the general population suggest that multiple pregnancies have higher risks of adverse outcomes [9, 101–105]. This should be kept in mind, especially for women receiving assisted-reproduction treatment, which is becoming increasingly popular with KT patients, and in turn creates a higher risk of adverse pregnancy-related outcomes [106–109].

Few studies have been specifically addressed to the long-term health of the children of KT mothers. Based on the scant evidence available, it appears that most children of KT mothers attain normal developmental goals. However, the neurological problems linked to prematurity should not be underestimated and must be explained at counselling [73, 78–94, 110–113]. The Study Group feels there is a clear need for further long-term studies focussing on the psychosocial health of these children and on their intellectual development.

## Which patients are the “best candidates” for pregnancy after KT and which patients are not?

(i) The following are the requirements most often cited for identifying the best candidates for pregnancy after KT from the clinical point of view:
  - Normal or good kidney function (differently defined: usually as above 60 ml/min)
  - No proteinuria or scarce proteinuria (differently defined: usually as below 300–500 mg/day)
  - No hypertension or well-controlled hypertension (the latter usually defined as treated in monotherapy and without organ damage)
  - Low-dose immunosuppression with “allowed” drugs
  - At least 2 years after KT (this interval has recently been reduced to 1 year after KT) (strong suggestion, several sources of observational data).
(ii) Further clinical maternal elements that can contribute to identifying the “best candidates” include:
  - No recent rejection episode
  - No recurrent urinary tract infection
  - Discontinuation of potentially teratogen drugs for at least 6 weeks (moderate suggestion, several sources of observational data and indirect evidence).
(iii) From the obstetric point of view, besides the absence of hypertension, and kidney disease, a low-risk mother is young (under 35), non-obese, non-diabetic, with a spontaneous singleton pregnancy (strong suggestion, several sources of observational data and indirect evidence)
(iv) All other cases are high-risk KT pregnancies. No graduation of risks is presently available (strong suggestion, several sources of observational data and indirect evidence).

Identification of the best candidates for pregnancy after KT is based on the results of observational studies [17, 32]. Overall, KT pregnancy risks display behaviours similar to those found in CKD pregnancies, and the best results are in patients with normal kidney function, normotension and no proteinuria [9, 17, 30, 32, 51, 56, 114–120].

On account of its patients’ characteristics, the transplant world has remained impermeable to the CKD staging system and the definition of “normal” kidney function is usually based upon serum creatinine, with different cut-points in different studies. The relationship between kidney function and pregnancy outcomes is not surprising, and is probably the first item to take into account in a risk assessment. However, no graduation of risks is available and the Study Group suggests, in the absence of new data, applying the same criteria that have proven to be efficacious in CKD (stratification along the classic CKD stages) [9, 11, 30, 56, 121].

Hypertension and proteinuria are identified as independent risk factors for adverse pregnancy outcomes [30]. However, as no study has tested their combined effect, the Study Group suggests basing counselling on the wider set of data on CKD pregnancies, in which the presence of hypertension and proteinuria exert a multiplicative effect on the risks. KT patients with hypertension and proteinuria should therefore be informed of the high incidence of preterm delivery in CKD patients with both hypertension and proteinuria, even in those with normal or near normal kidney function [30, 50, 122].

Immunologic stability is another must. This includes being rejection-free for at least 6 months (some studies do not define the interval and in others the length of time ranges from 6 months to 1–2 years), and being treated with low-dose immunosuppression. There were several studies that indicated that pregnancy was safer at least 2 years after KT; however, recent studies suggest that waiting for 1 year can lead to superimposable results [123–128]. A few reports of pregnancies immediately after KT or of KT during the early weeks of pregnancy indicate that success is also possible in these cases, warning against systematic pregnancy termination in such occurrences [129, 130].

As previously stated, a GFR above 90 ml/min, a blood pressure at or below 130/80 mmHg and proteinuria inferior to 300 mg/dl clearly defines an ideal situation. A grey area includes GFR 60–90 ml/min, blood pressure below 140/90 mmHg and proteinuria below 500 mg/day [17–26, 56].

The Study Group therefore proposes an individualised evaluation in all these cases.

The relationship between acute rejection and pregnancy is complex. This is a situation which calls to mind the relationship between pregnancy and flares in systemic lupus erythematous: in the past flares were considered frequent, while when they were subsequently re-dimensioned, this led to abandoning the policy of administering bolus steroids at or immediately after delivery [9, 131].

A pregnant grafted woman has been defined as a complex chimera with at least three cell populations: her own ones, the donor organ, and the foetus. Previous pregnancies and blood transfusions may have added other cell populations. The tolerance system is complex and not all antigens induce a response; tolerance may be the result of exposure of particular antigens or complexes [132].

The postpartum period is also of pivotal importance: the acute loss of the placenta, which was a primary driver of tolerance, could theoretically trigger rejection, while the decrease of interleukin (IL)-10 and estrogen levels may counterbalance this effect [132, 133]. Acute kidney injury (AKI) is a relatively unexplored syndrome post-partum, and new data suggest in particular to pay attention to the use of anti-fibrinolytic agents [134].

While recent epidemiological studies are reassuring concerning the risk of rejection, the Study Group feels it is vital to take into account the clinical impression that acute rejection during pregnancy or in puerperium can be particularly aggressive. While more experience-based than evidence-based, this consideration should suggest maintaining a high level of attention in these delicate moments of a woman’s life [17, 23, 24, 26].

Urinary tract infections are frequent in KT pregnancies; once more, the recent literature is scant. However, considering the experience in CKD pregnancies, the Study Group suggests maintaining a policy of high attention, and performing a urinary culture at least twice monthly, as counselled for CKD women with a history of upper urinary tract infections, kidney malformations or kidney scars [9, 135, 136].

Table 1 shows the main immunosuppressive drugs employed in pregnant KT patients. Azathioprine, cyclosporine, tacrolimus and steroids are considered safe [137–154]. Little is known about m-Tor inhibitors, but since they are teratogen in animals, they are avoided in humans, even if a few cases with a positive outcome have been recently reported in the literature [137–139]. Since mycophenolate can cause a characteristic embryopathy, known as MMF foetal syndrome, this drug should be discontinued at least 6 weeks before conception [123, 125, 140–152].

**Table 1:** Main immunosuppressive drugs for chronic treatment in pregnant KT patients (modified from reference [9])

Drug	Main features	FDA rating
Usually considered as safe		
Azathioprine	This is the most widely used immunosuppressive drug. It is teratogen in animal models, but not in humans, possibly because the foetal liver is not able to activate the drug. KDIGO and European Best Practice Guidelines suggest switching from mycophenolate to azathioprine before pregnancy	D
Cyclosporine A	This calcineurin inhibitor has not been associated with increased teratogenicity; however, small for gestational age babies and preterm delivery have been reported, possibly due to the maternal disease and not specifically to the drug. Levels may vary in pregnancy and the hypertensive, hyperglycaemic and nephrotoxic effects should be mentioned	C
Tacrolimus	The drug has similar effects and side effects to cyclosporine A; experience is more limited than with the previous drug	C
Steroids	Together with azathioprine these are the most often employed and best known drugs. The most frequently used short-acting corticosteroids include prednisone, methylprednisolone and prednisolone, while betamethasone and dexamethasone are among the long-acting drugs. No major malformations have been reported, and the issue of labiopalatoschisis is debated. A higher risk of premature rupture of membranes has been reported. Other relevant side effects include infectious risk, and the increased risk of gestational diabetes	C
To be avoided		
Mycophenolate	Severe foetal malformations are reported, mainly involving cardiovascular and cranial malformations. Discontinuation for at lest 6 weeks, to stabilize kidney function, is usually indicated after kidney transplantation	D
m-Tor inhibitors	Very few studies have considered their use in pregnancy. They are teratogenic in animals and discontinuation in humans is a matter of debate. KDIGO guidelines suggest discontinuation in anticipation of pregnancy	C
Rituximab, simulect	Too few studies to allow safe use in pregnancy. Need for further evidence, but trials are unlikely to be undertaken	C, D

FDA rating [135]: A, controlled human studies show no risk; B, no evidence of risk in studies; C, risk cannot be ruled out; D, positive evidence of risk; X, contraindicated in pregnancy

*KT* kidney transplantation, *FDA* US Food & Drug Administration, *KDIGO* kidney disease-improving global outcomes

While several studies focus on the effect of kidney function on pregnancy outcomes, less is known on the additive effect of the classical risk factors on KT pregnancies [153].

High maternal age increases the risk for preterm delivery and PE. Obesity is associated with a higher risk of the hypertensive disorders of pregnancy; the same is true for diabetes, which is also associated with congenital malformations, often at the cardiac level [17, 49, 154–156]. The risks are higher in type-1 diabetic mothers. The effect of assisted fertilization and multiple pregnancies, also associated with adverse pregnancy outcomes, will be further discussed below [157–159].

Acknowledging these knowledge gaps, the Study Group underlines the need for further studies targeting pregnancies in “non-ideal” KT pregnancies.

## Frequency and modality of controls in KT pregnancy

Follow-up should be intensified in KT patients as compared with normal pregnancies (strong recommendation, indirect evidence)

The main goals of follow-up are early identification and treatment of complications, including acute rejection, hypertension, anaemia, coagulation disorders, and timely planning of delivery (strong recommendation, indirect evidence)

No validated formula for GFR calculation in KT pregnancy is available, hence GFR should be assessed by 24-h urine collection (strong recommendation, based on GFR in normal pregnancy and PE)

By analogy with follow-up in pre-RRT CKD stages, follow-up should be intensified in KT patients with reduced kidney function, hypertension and proteinuria (strong recommendation, indirect evidence).

Follow-up should include at least one nephrology consultation with blood and urinary tests every 2–4 weeks in non-proteinuric, non-hypertensive KT patients with “good” kidney function; the frequency of controls should be increased in the case of proteinuria, hypertension or reduced kidney function (strong recommendation, indirect evidence).

Pregnancy after KT should be considered as “at high risk”, even in the presence of the “ideal” profiles delineated above, and the Study Group suggests increasing the frequency of controls in proportion to the decrease in kidney function, based on the presence of hypertension, proteinuria, urinary tract infection or any other risk factor; particular attention should be paid to the side effects of antirejection therapies (Table 1).

**Fig. 1:**
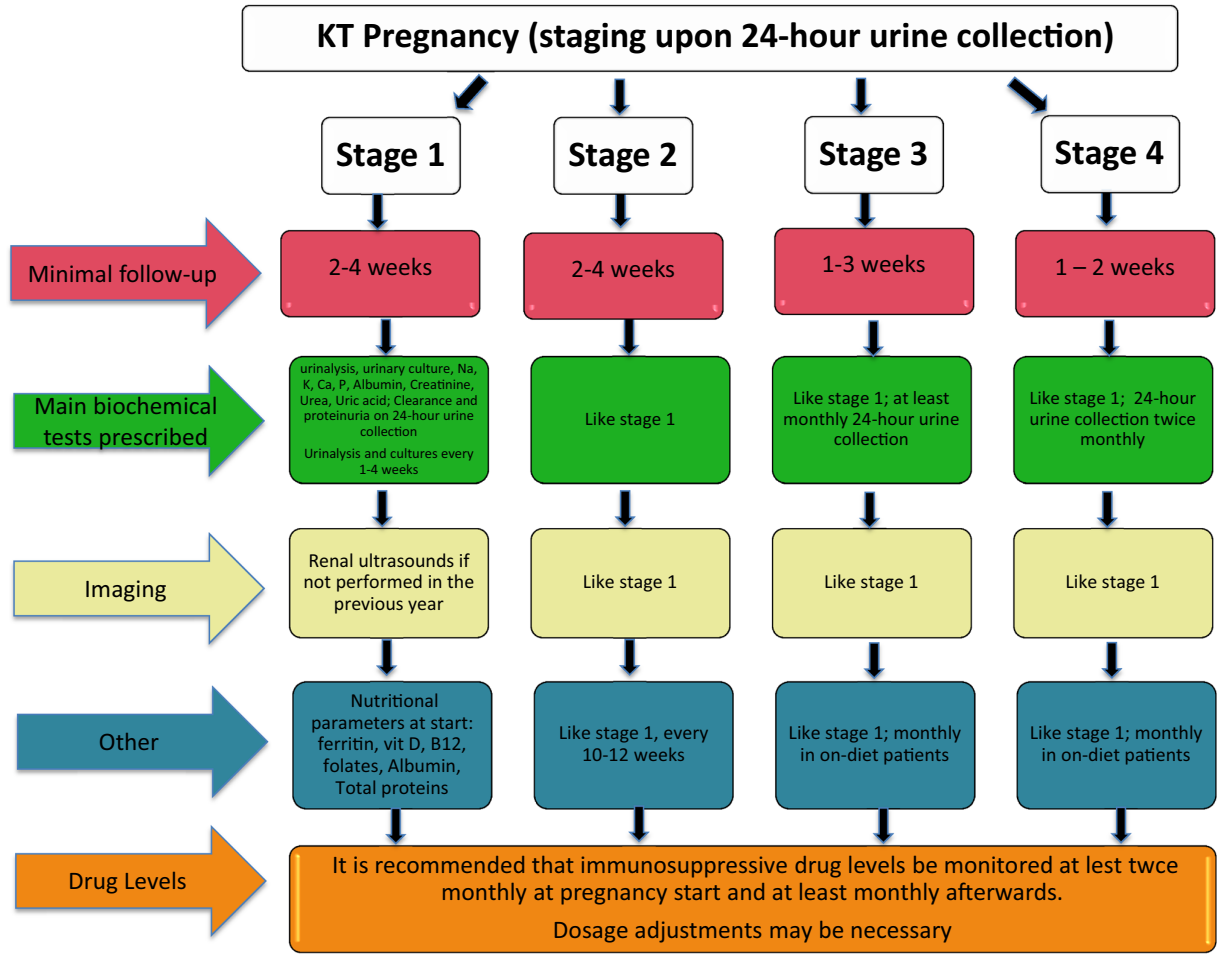
Proposed frequency of controls during pregnancy according to CKD stages in women with a kidney transplantation

The flow charts, adapted from the position statement on CKD and pregnancy, summarize the proposed frequency of controls (Fig. 1) [9].

The Study Group suggests a minimum requirement of one nephrology control with blood and urinary tests every 2–4 weeks in patients with “good” kidney function (in the absence of clear definitions, we consider this group to be women with at least 60 ml/min of creatinine clearance measured on 24-h urine collection), and without proteinuria and hypertension, increasing to weekly controls in patients with any combination of the risk factors mentioned above.

The assessment of kidney function is especially challenging in KT pregnancies. Little is known about the role of hyperfiltration, an increase in plasma volume and in volume distribution; validated GFR formulae are not available (a problem shared by all CKD stages), and creatinine clearance on 24-h urine collection is not frequently employed; similar considerations apply to proteinuria [160, 161]. Therefore, in the absence of agreed assessments, the Study Group considers it wise to refer to the same gold standard identified for CKD patients (based on 24-h urine collection for both creatinine clearance and proteinuria).

An important issue is probably also the presence of the physiological decrease or of an increase of serum creatinine in pregnancy. Once more, there are no specific data in this regard, but according to the clinical experience the Study group underlines the importance of paying attention to the presence-absence of physiological increase in the kidney function as a potential predictive element of pregnancy outcomes after KT.

The issue of an increase in extracellular volume is important and is probably one of the reasons for the frequent need to increase doses of the calcineurin inhibitors. The higher need for anti-rejection drugs is probably multifactorial as many patients need to at least double the initial doses prescribed, despite the fact that distribution volume is usually only about 30% higher (Table 1).

KT patients are at risk for urinary tract infections; therefore, urinalysis and urinary cultures should be a part of routine controls, and the frequency should be increased to weekly in patients with recurrent urinary tract infections during or before pregnancy (Fig. 1).

These considerations balance time-consuming assessments (24-h urine collection, frequent urine and/or blood tests) with the need for close control of the mother, foetus and grafted kidney, using simple, non-invasive procedures. The Study Group underlines that these recommendations are based mainly on scattered evidence and common clinical understanding. Further economic evaluations and organisational analyses are highly recommended.

## Treatment of hypertension in KT pregnancy

Pre-existing hypertension should be managed like chronic hypertension in CKD women, with careful monitoring, avoiding overcorrection (strong recommendation, indirect evidence and scattered data).

Hypertension occurring in pregnancy, with or without proteinuria, should be differentiated from PE, on account of the different prognosis for the two conditions in pregnancy (moderate recommendation, indirect evidence and scattered data).

Low-dose acetylsalicylic acid can be employed in the prevention of PE, in patients without contraindications, as in other high-risk pregnancies (moderate recommendation, indirect evidence and scattered data).

Blood pressure targets in hypertensive KT pregnancies have not been established, similarly to what was observed in CKD pregnancies [9, 13]. Learning from data available on pregnancies in chronic hypertension, overcorrection should be avoided, given its detrimental effects on foetal growth [162–171].

In keeping with the indications for CKD pregnancies, on the basis of the results of the large Control of Hypertension In Pregnancy Study (CHIPS) trial, our group suggests implementing strict blood pressure control (“ideal” target < 130/80 mmHg, acceptable < 140/90 mmHg), under careful clinical surveillance [9, 168].

As reported in Table 2, no anti-hypertensive drug is labelled as fully safe in pregnancy. Most importantly, the teratogenicity of angiotensin-converting enzyme inhibitors (ACEi) and angiotensin II receptor blockers (ARBs) is still a matter of debate; therefore, the Study Group strongly supports pre-emptive discontinuation of ACEi and ARBs in patients without proteinuria, and early discontinuation at the first positive pregnancy test (4th–6th gestational week) in patients in whom proteinuria is considered a potential threat for kidney function [9, 171–176].

**Table 2:** Commonly needed drugs in pregnant patients with kidney transplantation (modified from reference [9])

Drug	Anti-hypertensives	FDA rating
Usually considered first choice		
Alpha-methyl dopa	Widely used, with no reported negative effects on the foetus or on its subsequent development. May not be able to correct severe hypertension	B
Niphedipine	The long-acting drug most commonly used in pregnancy. The increase in peripheral oedema may be a relevant side effect in CKD patients	C
Labetalole	Usually well tolerated, should be avoided in subjects with asthma. In a RCT it was shown to be comparable to alpha-methyldopa	C
Usually considered second choice		
Beta blockers	The main drawback was foetal growth restriction. Atenolol (D) often involved. May be effective in severe hypertension. May induce hypoglycaemia, hypotension and bradycardia at delivery	B Pindolol C Metoprolol D Atenolol
Clonidine	Side effects and rebounds at discontinuation are common. Slowing foetal growth also reported	C
Alpha blockers	Other drugs should be preferred since controlled studies are missing	C
Diuretics	Usually avoided. Thiazides may be continued. Amiloride may be employed in Gitelman syndrome	B Hydrochlorothiazide Amiloride
To be avoided		
Short-acting niphedipine	Contraindicated by FDA, RCOG and AIPE due to the risk of severe sudden hypotension with detrimental effects on placental flows	D
ACEi ARBs	Risk of major malformations, in particular in the second and third trimester	C 1st trimester D 2nd 3rd trimester

FDA rating: A, controlled human studies show no risk; B, no evidence of risk in studies; C, risk cannot be ruled out; D, positive evidence of risk; X, contraindicated in pregnancy

*RCOG* Royal College of Obstetricians and Gynaecologists, *AIPE* Associazione Italiana Preeclampsia, *ACEi* angiotensin-converting enzyme inhibitors, *ARBs* angiotensin II receptor blockers. For other abbreviations, see Table 1

The differential diagnosis between hypertension and proteinuria due to recurrence of the original disease, chronic allograft nephropathy or reduced nephron mass and PE is clinically important, since the maternal-foetal prognosis is worse in the latter [177–179].

Uric acid levels are well known markers of risk of adverse pregnancy outcomes in pregnancies in the overall population; there are limited data after kidney transplantation, suggesting that low pre-conception uric acid levels are correlated with positive pregnancy outcomes, but their role as biomarkers is not confirmed after kidney transplantation [180].

Among the biomarkers of PE, the ratio between soluble Fms-like tyrosine kinase 1 (sFlt-1), a receptor for both vascular endothelial growth factor (VEGF) and placental growth factor (PIGF) is of high interest at least in ruling out the severe forms of PE [181–184]. The role of these biomarkers in the differential diagnosis between PE and CKD is still under study, and the Study Group recommends using the test, when available, in support of clinical reasoning [9].

## Management of proteinuria in KT pregnancy

Evidence on pregnancy in KT patients with proteinuria is limited, hence the main suggestions are derived from the broader experience with CKD patients (medium recommendation, indirect evidence)

Low-dose acetyl salicylate is indicated in proteinuric patients (as well as in patients with reduced kidney function or hypertension) (strong recommendation, different levels of evidence in various diseases)

Efforts should be made to counterbalance hyperfiltration: while albumin infusion should be avoided, moderate protein restriction can be attempted (medium recommendation, indirect evidence)

Low-dose acetyl salicylate (ASA) for the prevention of PE is suggested by a large body of evidence; all KT patients share an increased risk of PE, higher in patients with proteinuria or hypertension [185–193]. The best timing for starting ASA is debated; the Study Group suggests that ASA should be continued in pregnancy if already part of the chronic treatment. In the case of ASA start, early start (at positive test) to favour placentation or later start (2nd trimester) to minimise bleeding risks in the case of miscarriage, should be discussed within the multidisciplinary team [9].

Two recent meta-analyses suggest that early ASA use (start before 16 weeks) is associated with better outcomes, but the dose is different in the two studies and the effect is only significant as for prevention of early PE, which is probably less frequent in transplanted, as well as CKD women, than “late-maternal” PE [192, 193].

Due to the pathophysiological importance of hyperfiltration in long-term kidney damage, the Study Group suggests avoiding, whenever possible, treatments which potentially increase hyperfiltration, such as albumin infusion [194].

Although we lack robust evidence on the advantages of a moderate protein restriction, often suggested in other forms of CKD, the Study Group recommends avoiding protein-rich diets, and considering the potential of this nutritional approach at least in cases in which hyperfiltration is thought to play an important role, in keeping with what is advised in CKD [9, 195, 196].

## Management of KT pregnancy with reduced kidney function

Evidence on pregnancy in KT patients with reduced kidney function is scant, probably as a result of the policy of discouraging pregnancy in this context (medium recommendation, indirect evidence)

Given the lack of data grading the effect of reduced kidney function in KT pregnancy, counselling and management should be based on the experience in CKD (strong recommendation, indirect evidence)

There is no indication on when to start dialysis in pregnancy in the setting of a failing KT. This knowledge gap should be considered in counselling (strong recommendation, indirect evidence).

Evidence on pregnancy when there is a failing KT is extremely limited, differently from the growing body of evidence on pregnancy in the most advanced CKD stages in native kidneys. The most likely explanation is that this is an indirect effect of discouraging pregnancy in “less than optimal” conditions after KT [197].

As previously discussed from the bioethical point of view, the growing importance attributed to patients’ autonomy will probably modify this situation, but our lack of data makes precise counselling difficult, and points to basing advice upon the larger body of data available on native kidneys. In this regard, the Study Group would like to refer to the previous “best practice” on pregnancy in CKD [9].

A particular aspect, not fully clarified even in non-pregnant KT patients, regards when to restart dialysis in a failing KT. Bearing in mind this lack of evidence and acknowledging the experience in the non-pregnant population and the most recent guidelines, the Study Group suggests adopting individualised policies, based upon clinical judgement and not on GFR only, in agreement with what was previously discussed in CKD [9].

## Management of inadvertent pregnancy under potentially teratogen agents, or undertaken “too early”

The most important potentially teratogen agent employed after KT is mycophenolate mofetil (MMF), whose use is associated with severe malformations, mainly skeletal and ocular ones, and with mental retardation (strong recommendation, scattered data)

The severity of the syndrome is variable and MMF treatment is also compatible with apparently normal development. The reasons for the syndrome’s different presentation are not known (strong recommendation, scattered data)

Physicians of women under MMF should be aware that they need to interrupt treatment at least 6 weeks before a patient becomes pregnant (strong recommendation, scattered data)

Other potentially teratogen drugs (m-TOR inhibitors, ACEi and ARBs) have less clear risk patterns and probably have a lower incidence of malformations (strong recommendation, scattered data)

The issue of pregnancy occurring “too early” merges the frequent use of contraindicated drugs and higher immunological risks (strong recommendation, scattered data)

The caregiver multidisciplinary team should support the patient’s choice to continue or terminate pregnancy, offering careful counselling and making use of imaging to detect major foetal abnormalities (strong recommendation, indirect data)

When pregnancy is undertaken early in the course of transplantation or is unexpected, the use of drugs that are contraindicated (such as MMF), or for which there is a reasonable doubt of teratogenicity (such as ACEi, ARBs and m-TOR inhibitors), poses clinical and ethical problems. The issue of timing for discontinuation before elective pregnancy is still open; consensus is now on discontinuation of MMF and enteric coated mycophenolate sodium (EC-MPS) at least 6 weeks before attempting conception; mTOR inhibitors should also be discontinued or replaced before pregnancy is attempted, even if positive reports are available (Table 1).

The most important problem occurs when pregnancy is unexpected and conception has occurred while the patient was on these treatments; in fact, none of these drugs is a cause of malformations in 100% of cases, thus leaving space for individual choice. While ultrasounds are increasingly able to detect major skeletal and organ malformations, some relevant malformation is not detectable, or is first seen in phases in which pregnancy interruption is no longer possible [198].

In the absence of potentially teratogen drugs, the classic contraindication to pregnancy in the first 2 years after renal transplantation is based upon the clinical assumption that the risk of acute rejection, graft dysfunction, and infection are higher than in the subsequent period. The empiric limit of 2 years has recently been challenged, suggesting that outcomes may level after the first year of KT, thus widening the “ideal” timing, also taking into account that kidney function is usually better in the first years after kidney transplantation [125, 126].

In this regard, the Study Group suggests that conception should be avoided in the first year after KT. The best contraception treatment is still a matter of debate and a further best-practice paper will be dedicated to this issue. In the lack of evidence, the Study Group suggests a tailored approach be adopted, taking age, kidney function, risk of infection, renal disease and coagulation derangements into account [128, 199–203].

In the case of “early” conception, i.e. in the first year after KT, in the absence of teratogen drugs, the Study Group does not support a policy of systematic pregnancy interruption, but once more suggests a personalised policy that considers the increased risk of graft dysfunction, and the likelihood of there being a further pregnancy in a “safer” period, based on considerations such as the patient’s age and, if possible, information on her ovarian reserve.

## Management of KT patients with multiple gestations

Multiple gestations, whether spontaneous or occurring as a result of assisted fertilization procedures, are at higher risk for adverse pregnancy-related outcomes (strong recommendation, indirect data)

There are few data on multiple pregnancies after KT and the ones we have are probably affected by reporting and publication biases. Within these limits, it seems that good outcomes are possible and may be the rule (medium recommendation, indirect data)

There is no need to interrupt multiple-gestation pregnancy in KT; however, follow-up should be intensified and an expert on the clinical management of multiple gestations should be included in the team (strong recommendation, indirect data)

In the general population, multiple gestations, whether spontaneous or occurring as a result of assisted fertilization procedures, are at higher risk for adverse outcomes [101, 157–159]. A similar increase in risk has been described in pregnancies occurring in CKD patients, although the amount of data is limited, and there is probably a reporting and publication bias, leading to over reporting of favourable outcomes with respect to unfavourable ones. The current evidence only allows us to state that good outcomes are possible and may even be the rule, thus indicating that there is no reason for pregnancy termination in “ideal candidates”. However, follow-up should be intensified and an expert on the clinical management of multiple gestations should be included in the team. Patients in less than ideal situations should be warned both about the absence of elements allowing precise risk assessment, and the fact that outcomes are less favourable in multiple pregnancies, whether or not these have resulted from assisted fertilization procedures [101–103, 105].

## Advising KT patients who want to undergo assisted fertilization procedures

Assisted fertilization in the general population has a higher risk of PE, intrauterine growth restriction, and prematurity (strong recommendation, observational data)

Assisted fertilization procedures are feasible in KT patients, but the evidence is scant and affected by reporting and publication biases. Within these limits, good outcomes are reported virtually with all techniques, but the incidence of adverse events is not known (strong recommendation, indirect data)

In the case of pregnancy after assisted fertilization techniques, follow-up should be intensified and experts should be included in the team (strong recommendation, indirect data)

Assisted fertilization is increasingly chosen in the general population, to overcome the limits imposed by age and chronic diseases. All these techniques are associated with higher risk of PE, intrauterine growth restriction, and prematurity; the risks increase with the need for in vitro manoeuvres, reaching a peak for egg donation. According to an increasing number of reports, mainly based on single cases, assisted fertilization procedures are feasible in KT patients, but the evidence is scant and there are likely to be important reporting and publication biases, leading to over-reporting of successful cases [106–108]. Within these limits, good outcomes after KT are reported when using virtually all techniques, but the incidence of adverse events is not quantifiable on the basis of the present state of evidence, as only one relatively large series was found by our extensive search strategy [108].

Since assisted fertilization techniques are associated with a significant increase in adverse events in the general population, the Study Group urges that follow-up be intensified and experts in the field be included in the multidisciplinary team in KT pregnancy after assisted fertilization.

While, given the present state of the art, the Study Group does not feel that nephrologists should encourage or discourage these procedures, our position is that while we support the choice of the mother-to-be, we should counsel techniques that are more likely to lead to a single rather than to a multiple pregnancy. Due to the complexity of this problem, the Study Group plans to discuss it in a further dedicated best-practice review.

## Pregnancy in kidney-pancreas transplantation: specific issues

Solid-organ-transplant recipients should be considered high-risk patients for pregnancy (strong recommendation, indirect data).

In pancreas-kidney transplantation (PKT) the presence of two organs in the pelvis poses additional risk to both mother and newborn (strong recommendation, indirect data).

The pancreas graft probably presents fewer complications during pregnancy in the presence of normoglycaemia (medium recommendation, scant data).

Birth weight and gestational age are usually lower when compared to KT (strong recommendation, registry data).

An integrated multidisciplinary team including a transplant physician, nephrologist, diabetologist, and obstetrician is essential in counselling and follow-up (strong recommendation, indirect data).

In the case of PKT, as in all solid-organ transplantation, recipients should be considered high-risk for pregnancy and management should be carried out by a multidisciplinary team, including a diabetologist, even in the presence of normal glycaemic levels. In fact, in PKT recipients the presence of two organs in the pelvis poses an additional risk to both mother and newborn [8, 204]. The risks are miscarriage, preterm birth, foetal malformation, preeclampsia, deterioration of renal function, and pancreatitis of the graft, reported even after uncomplicated delivery [205–207].

The pancreas graft probably presents fewer complications during pregnancy in the presence of normoglycaemia and, as in all cases of solid-organ transplantation, the risks are reduced if the pregnancy is planned, and if the organs are functional. As in the case of KT, the suggested timing is at least 1 year after transplantation with normal blood pressure, absent proteinuria and stable doses of immunosuppressive drugs.

Registry data indicate that mean birth weight and gestational age are lower when compared to kidney-only recipients, but the reasons for this are still a matter of debate [207].

For these reasons, the Study Group suggests that pregnant PKT mothers should be monitored very closely, indicatively every 2 weeks, during pregnancy. A fully integrated multidisciplinary team, including a transplant physician, nephrologist, diabetologist, and obstetrician is essential for counselling and in the follow-up of PKT pregnancy.

## Working conclusions

Pregnancy is an added value for women with kidney transplantation; fertility is at least partly restored and a successful pregnancy is possible, frequent, and usually successful. However, KT pregnancies have a higher risk of PE, preterm delivery and small for gestational age babies than pregnancies in the general population.

The profile of the “best” candidate for pregnancy after KT is relatively well drawn (normal or “good” kidney function, usually considered as a GFR above 60 ml/min), normotension or well controlled hypertension (one drug only, in the absence of end-organ damage), no proteinuria (or proteinuria below 300–500 mg/day), no recent rejection episode, and conception at least 1 year after KT. Definitions are however not always univocal, and less is known about “non-ideal” candidates, who pose important clinical and ethical problems in the complex interaction between the “best choice” for the mother, for the child and for kidney function.

Thanks to the progress in KT therapies, to improvements in the management of high-risk pregnancies, to the growing importance of patients’ self-determination, and to new options, such as assisted fertilization, the panorama of pregnancy after KT is changing.

The side effect of this positive outlook is that evidence is heterogeneous and sometimes difficult to interpret. This lack of certitudes should be borne in mind at counselling, and strongly supports shared choices in the context of multidisciplinary care, as well as individualised approaches. Nephrologists need continuous updating in this emerging field, and once more the Study Group supports the establishment of educational programs and of multicentre research studies.
